# Supplementary material for: Drug-induced vitiligo: a real-world pharmacovigilance analysis of the FAERS database
Source: PLoS One. 2025 Sep 8;20(9):e0332079. doi: 10.1371/journal.pone.0332079 (PMC12416669; doi:10.1371/journal.pone.0332079)
Supplement: S1 Table — (DOCX) [file pone.0332079.s001.docx]

**Supplementary information**

S1 Table. Algorithms Employed for Signal Detection

| Algorithms | Equation | Criteria |
| --- | --- | --- |
| ROR | ROR = ad/b/c | - lower limit of 95% - CI>1, N≥3 |
|  | 95%CI = e^ln(ROR)±1.96(1/a+1/b+1/c+1/d)^0.5^ |  |
| BCPNN | IC = log_2_a(a+b+c+d)(a+c)(a+b) | - IC_025_>0 |
|  | $\gamma= \gamma_{\mathrm{ij}}\frac{(N+\alpha)(N+\beta)}{(a+b+\alpha_{i})(a+c+\beta_{j})}$ |  |
|  | $E(IC)=\log_{2}(\frac{(a+\gamma_{\mathrm{ij}})(N+\alpha)(N+\beta)}{(N+\gamma)(a+b+\alpha_{i})(a+c+\beta_{j})})$ |  |
|  | $V\left( IC \right)=\frac{1}{{(log2)}^{2}}\left[ \frac{N-a+\gamma-\gamma_{\mathrm{ij}}}{\left( a+\gamma_{\mathrm{ij}} \right)\left( 1+N+\gamma\right)} +\frac{N-a-b+\alpha-\alpha_{i}}{(a+b+\alpha_{i})(1+N+\alpha)}+\frac{N-a-c+\beta-\beta_{j}}{(a+c+\beta_{j})(1+N+\beta)} \right]$ |  |
|  | $IC025= E(IC)－2\sqrt{V(IC)}$ |  |

a: number of vitiligo cases to the target drug; b: number of other adverse event cases to the target drug; c: number of vitiligo cases to background drugs; d: number of other adverse event cases to the background drug; N: the number of total cases.

CI, confidence interval; N, the number of reports; IC, information component; IC_025_, the lower 95% CI for the IC; E(IC), the IC expectations; V(IC), the variance of IC; BCPNN, Bayesian confidence propagation neural network; ROR, reporting odds ratio.
