# Supplementary material for: Drug-induced vitiligo: a real-world pharmacovigilance analysis of the FAERS database
Source: PLoS One. 2025 Sep 8;20(9):e0332079. doi: 10.1371/journal.pone.0332079 (PMC12416669; doi:10.1371/journal.pone.0332079)
Supplement: Attachment — Submitted filename: Response letter.docx [file pone.0332079.s002.docx]

Response Letter

Dear Dr. Florie Lacorte Canales,

Thank you for your feedback on our manuscript (Manuscript Number**:** PONE-D-25-31175). We have carefully addressed the requirements regarding the Human Participants Research Checklist and hereby provide the following responses:

**1. Ethics approval confirmation**

- **Checklist response**: Marked "N/A" (no ethics approval required).
- **Rationale**: The study utilizes publicly available data from the FDA Adverse Event Reporting System (FAERS), which is de-identified and anonymized. As the research does not involve direct interaction with human participants or identifiable information, ethical approval is not required.

**2. Prospective recruitment of human participants**

- **Checklist response**: Marked "N/A".
- **Rationale**: The study is a retrospective pharmacovigilance analysis based on pre-existing FAERS data, and no participants were prospectively recruited (e.g., no clinical trials, questionnaires, or tissue sampling were conducted).

**3. Retrospective study details**

- **ⅰ. Data access period**:
  The FAERS database was accessed for research purposes from the first quarter of 2004 to the fourth quarter of 2024, as reported in the Methods section (line 79).
- **ⅱ. Identifiable information**:
  “During data collection, authors did not have access to information that could identify individual participants, as all FAERS data are de-identified and anonymized prior to public release.” This is stated in the Methods section (lines 89–92).

**Additional notes:**

- The completed Human Participants Research Checklist has been uploaded as a file type "Other" in the resubmission.

Please let us know if any further adjustments are needed. We appreciate your assistance in advancing this submission.

Kind regards,
Nonger Shen
