# Supplementary material for: Drug-induced vitiligo: a real-world pharmacovigilance analysis of the FAERS database
Source: PLoS One. 2025 Sep 8;20(9):e0332079. doi: 10.1371/journal.pone.0332079 (PMC12416669; doi:10.1371/journal.pone.0332079)
Supplement: Attachment — Submitted filename: Response for Data Availability Statement.docx [file pone.0332079.s003.docx]

Dear Dr. Descatamiento,

Thank you for your email and for the opportunity to clarify our Data Availability Statement. We confirm that there is no specific dataset title required to access the data used in our study. Our analysis encompassed all publicly available data quarters from the FDA Adverse Event Reporting System (FAERS) database, spanning from the first quarter of 2004 to the fourth quarter of 2024. The data is freely accessible to any user from the provided link without the need for a specific subset or accession number. This approach is consistent with the data availability methods employed in other pharmacovigilance studies previously published in PLOS ONE [1, 2].

As requested, we have updated the Data Availability Statement in our manuscript to the following: “The datasets utilized in this study are publicly available from the FAERS database online repository: <https://fis.fda.gov/extensions/FPD-QDE-FAERS/FPD-QDE-FAERS.html>. This analysis encompassed data from Q1 2004 to Q4 2024.”

Thank you again for your time and consideration. Should there be any further questions or required clarifications, please do not hesitate to contact us.

Sincerely,

Nonger Shen

Reference

1.	Ji H, Zhao Z, Zhao C. Unveiling Aducanumab's safety profile: A comprehensive pharmacovigilance analysis. PloS one. 2025;20(7):e0328076.

2.	Zhang C, Wang H, Chen X, Liu Y, Jiang P. Safety of lifitegrast: A real-world pharmacovigilance study based on FAERS. PloS one. 2025;20(4):e0321307.
